# A Novel Dynamic Growth Rod Inducing Spinal Growth Modulation for the Correction of Spinal Deformities

**DOI:** 10.1002/jsp2.70031

**Published:** 2025-01-10

**Authors:** Yangyang Xu, Da Lu, Le Zhang, Shijia Zhang, Yong Wu, Heng Li, Baoqing Pei, Xueqing Wu

**Affiliations:** ^1^ Beijing Key Laboratory for Design and Evaluation Technology of Advanced Implantable & Interventional Medical Devices, Beijing Advanced Innovation Center for Biomedical Engineering, School of Biological Science and Medical Engineering Beihang University Beijing China; ^2^ Foot and Ankle Surgery Department, Beijing Jishuitan Hospital Capital Medical University Beijing China

**Keywords:** biomechanical response, early‐onset scoliosis, fatigue, mechanical properties, novel growth rod

## Abstract

**Background:**

Growth rods are the gold standard for treating early‐onset scoliosis (EOS). However, current treatments with growth rods do not optimize spinal growth in EOS patients, and frequent distraction surgeries significantly increase complications, imposing considerable economic and psychological burdens on patients. An improved growth rod is urgently required to address the need for dynamic growth and external regulation.

**Methods:**

This study designed a novel growth rod (NGR) with unidirectional sliding and external regulation capabilities. By establishing a three‐dimensional model of the EOS spine, we simulated the implantation of traditional growth rods (TGR) and NGR. We applied a compressive load of 400 N to test axial stiffness and a moment of 1 NM to assess bending stiffness under six different conditions. Additionally, we evaluated the range of motion (ROM) of the spinal joints, and the distribution of Von Mises stress in vertebrae, intervertebral discs, and the growth rods, and calculated the axial force, moment, fatigue life, and strain energy of the device.

**Results:**

NGR exhibits higher axial compression and torsional stiffness than TGR and the Intact group. Additionally, Von Mises stress values for NGR are higher than those for TGR across all operating conditions, albeit with slightly lower total strain energy than TGR. Although Von Mises stress in NGR concentrates near the screw fixation, the fatigue life remains adequate for basic living requirements.

**Conclusion:**

Overall, NGR demonstrates superior stiffness and stress distribution. NGR's distraction‐based implant features a unidirectional sliding component with a spring‐driven mechanism for dynamic correction and a novel non‐invasive extension mechanism to reduce infections. Compared to leading EOS implants, NGR offers improved stability, showing promise for enhancing EOS surgical interventions.

## Graphical abstract

Our research addresses the limitations of current growth rod treatments for early‐onset scoliosis (EOS), particularly their failure to optimally promote spinal growth and the necessity for frequent distraction surgeries, which lead to significant complications and burdens on patients. We have developed a novel growth rod (NGR) featuring unidirectional sliding and external regulation capabilities. This design promises to improve clinical outcomes by providing dynamic correction functionality and reducing the risk of infection through a non‐invasive lengthening mechanism.

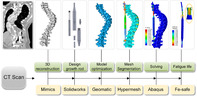

## 1 Background

The gold standard surgical treatment for early‐onset scoliosis (EOS) has traditionally involved deformity control through either single or dual‐growth rods [1]. However, these necessitate periodic lengthening, leading to numerous complications due to inadequate fixation and spontaneous spinal fusion [2, 3]. Managing severe or progressive spinal deformities in immature spines poses challenges. Surgical corrections of spinal curves and fusions in children with significant growth potential may adversely affect subsequent development. Besides the known risks of motion and loss of adjacent segment diseases with long‐segment fusions, loss of growth potential can significantly reduce attained height, potentially exerting severe negative impacts on pulmonary development, maturation, and function in children [4]. Extensive spinal fusion in a 5‐year‐old child forewarns a potential loss of vertical height by 12.5 cm: 7.8 cm in the thoracic spine and 4.7 cm in the lumbar segments [5]. Due to limitations in chest development, range of motion, and growth, traditional treatments such as casting, bracing, and spinal fusion have shown limited efficacy [6, 7, 8]. While spinal fusion remains a reliable treatment for correcting spinal curvature, it comes with potential negative consequences. Fusion surgery permanently restricts spinal movement in the fused segments, leading to reduced spinal range of motion (ROM) and an increased risk of adjacent‐level arthritis in patients [9, 10]. Therefore, recent interest has focused on new strategies for effectively managing severe infantile scoliosis surgically without resorting to multisegment spinal fusion. Growth rod treatment should be considered after failure of plaster/casting or bracing for most children with progressive curvature and young age. Given the array of issues encountered in the clinical management of EOS, there is considerable interest in employing growth‐friendly non‐fusion strategies in early‐stage spinal deformity management [1]. Recently, several non‐fusion treatment options based on the concept of “growth” have been developed to provide surgical intervention for EOS patients [11]. Adjustable‐length growth rods require ongoing manipulation to adjust to the patient's growth. Because these growth‐guided implants can slide at the rod‐screw junction, they can continuously adjust bone growth. The fundamental concept behind adjusting bone growth is altering the mechanical environment of the vertebrae and guiding their growth. In pioneering work by Stokes [12], the “Hueter–Volkmann Law” was demonstrated in a rat tail model, suggesting that the same concept could be applied to scoliosis to modulate growth. This law states compressive forces inhibit growth, while distractive forces stimulate it. By altering the mechanical environment to modulate growth, deformities can potentially be corrected [13, 14]. This essentially allows concave sides to regain growth velocity, gradually correcting the deformity. Samdani et al. [15, 16] have demonstrated that this growth modulation technique can progressively correct deformities without fusion. This essentially enables concave sides to regain growth velocity, correcting progressively the deformity. These growth‐friendly surgical approaches are widely classified as distraction‐based, growth‐guiding, or compression‐based treatments [17]. Common examples include conventional growth rods [6], magnetically controlled growth rods (MCGR) [18], the Luque Trolley growth rod system [19, 20], the Shilla Growth Guidance Technique [21], and the recently developed vertebral body tethering technique [22].

The traditional growth rod (TGR), as the earliest preserved growth technique for treating EOS, has the longest follow‐up time. However, TGR has numerous drawbacks. Typically employing fixed‐length designs, TGR cannot accommodate the changing spinal morphology of patients as they grow. Consequently, patients require frequent rod replacements throughout their growth, each necessitating surgery, thereby increasing surgical risks and patient discomfort. The inability to dynamically adjust growing rods may diminish spinal correction efficacy over time. Additionally, fixed‐length designs impede sustained corrective forces and impact treatment outcomes. The rigidity of growing rods may restrict natural spinal movement and growth. Repeated lengthening surgeries pose risks of recurrent anesthesia and increased infection rates from repeated wound openings. The burden of frequent surgical lengthening places a heavy strain on children and families, and the clinical consideration of surgery frequency and economic issues often leads to extended intervals between surgeries, significantly limiting the growth potential of EOS patients. Because of stability issues, complications associated with the TGR technique are notably high, ranging from 55% to 58% [7]. In recent years, the global application of MCGR, which allows for non‐invasive lengthening, has increased. Early results from several studies are promising, presenting systematic efficacy and cost‐effectiveness [3, 23, 24, 25, 26]. The US Food and Drug Administration approved this technology in the United States in 2014. One of the system's drawbacks is the relatively high initial cost of the magnetic rods. Although it effectively avoids the issues associated with multiple anesthetics, its ability to maintain distraction over time, the impact of metal debris deposition on adjacent soft tissues, implant failure, and changes in serum metal ion concentrations all pose unique challenges [27, 28, 29], with MCGR failure leading to increased surgical frequencies being particularly severe [30, 31, 32, 33]. Currently, TGR remains one of the most popular and widely used methods.

In response to the current status of growing rod devices, this study has designed a novel growth rod (NGR) that enables external adjustment, adapting to the growth process effectively. The innovative adjustable growing rod can dynamically adapt to the patient's growth status, providing continuous corrective force. This advancement is poised to significantly enhance the treatment outcomes of scoliosis, reducing the incidence of complications. By decreasing the frequency of rod replacements and surgical procedures, the new adjustable growing rod can markedly reduce patients' surgical trauma and postoperative recovery time. The application of minimally invasive surgical techniques can further mitigate surgical risks and patient discomfort. The flexible design and dynamic adjustment capability of the NGR can diminish patients' discomfort in daily activities, enhancing their ROM and quality of life. Patients can maintain a higher quality of life during treatment, alleviating psychological burdens. By reducing the number of surgeries and improving treatment effectiveness, the NGR can lower overall medical costs. For families of patients requiring long‐term treatment, this will significantly alleviate financial burdens. This study compares the performance of the NGR with TGR in terms of axial stiffness, bending stiffness, post‐implant spinal ROM, axial force, bending moment, the distribution of Von Mises stress in vertebrae, intervertebral discs and the growth rods, strain energy, and fatigue life. By amalgamating the usage guidelines of the NGR, a prospective, realizable diagnostic and therapeutic plan is conceptualized to offer superior treatment modalities for EOS, enhancing treatment efficacy.

## 2 Design Specification

To enhance the clinical success of the NGR design, it is crucial to conduct mechanical performance testing on the device. The testing protocol should be clinically relevant, aiming to predict failure loads and locations that may be observed in patients. This protocol also needs to be sufficiently straightforward to provide a basic assessment of implant strength [34]. Current standards such as ASTM‐F1717 [35] and ISO‐12189 [36] have been revised to better represent the true performance of spinal posterior implants [37, 38, 39, 40, 41, 42].

1. The effective lengthening distance of the device ranges from 40 to 80 mm, depending on the length of the core rod. This considers the maximum average spinal distraction observed during treatment, which varies from 46.7 mm [6] to 57 mm [43] within the distraction zone.
2. The main structure of the sleeve is cylindrical, while the core rod is rectangular, providing anti‐rotation functionality. The titanium rod has a diameter of 5 mm, and the sleeve has a diameter of 14 mm, which prevents the implant from protruding above the patient's skin surface.
3. The novel device provides manual distraction and incorporates metallic protrusions as reference points, enabling the surgeon to visually monitor the distraction distance in real time. This allows for precise control during the lengthening procedure.
4. This approach effectively reduces the number of surgical procedures. Patients can undergo distraction procedures in an outpatient setting, with follow‐up and distraction combined into a single process, thereby achieving external adjustment functionality.
5. Reducing the incidence of rod breakage complications is crucial. Rod fractures are caused by fatigue bending and compressive loads occurring during the patient's flexion movements [44]. The implant should provide fatigue strength under supraphysiological loads, tested over 5 million cycles at 5 Hz [45]. The novel device should aim to improve upon this benchmark.
6. The novel device provides a maximum length of 180–220 mm, matching the thoracic height required at skeletal maturity to ensure adequate respiratory function [46].
7. Manufactured from biocompatible materials.
8. Designed as a single rod, its intended use can, however, involve a pair of rods depending on the patient's needs and the surgeon's assessment.

## 3 Materials and Methods

### 3.1 Description of the Device

**FIGURE 1:**
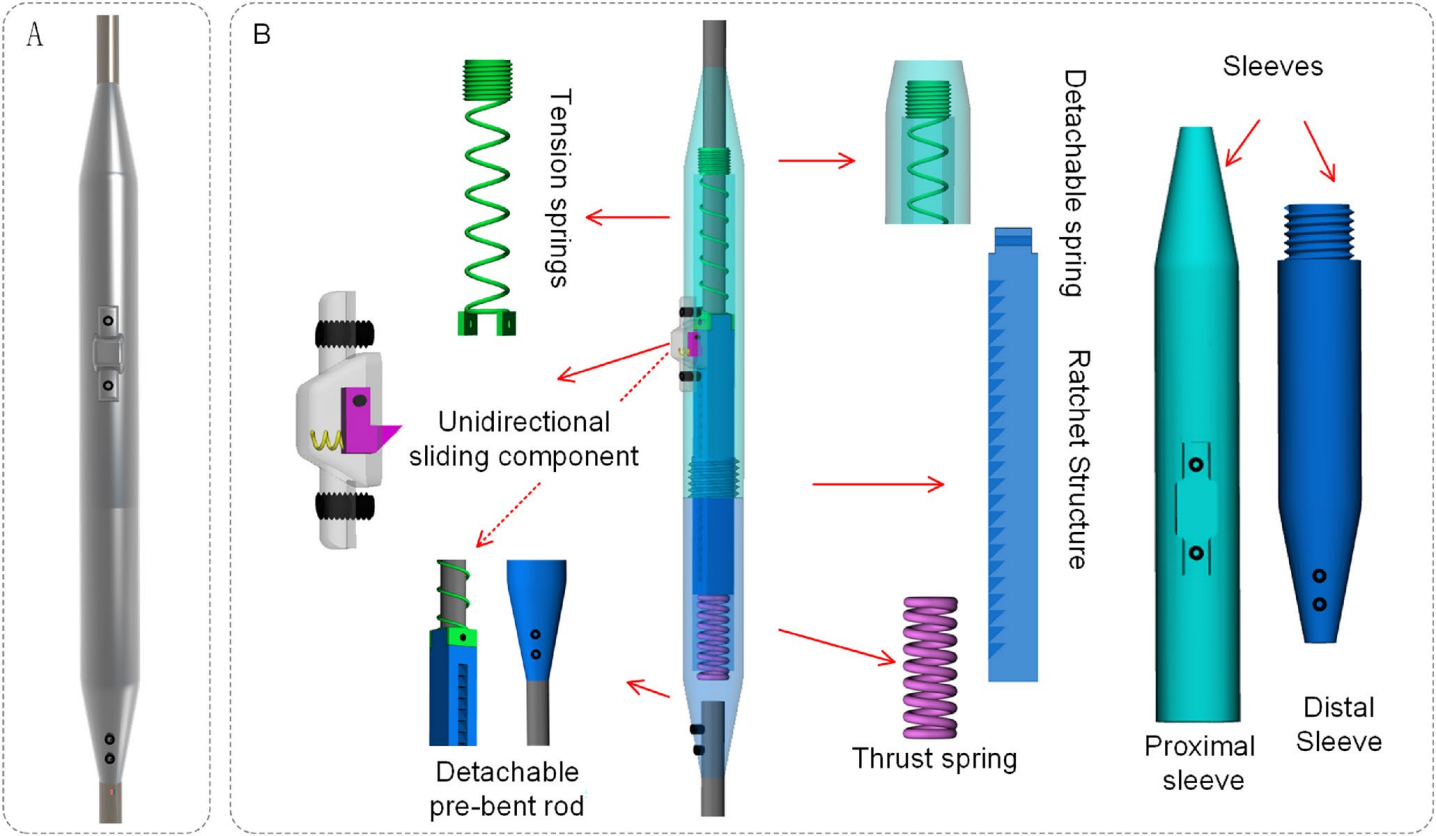
NGR structure diagram. (A) Model; (B) Structural details.

The NGR device comprises a main sleeve (including a proximal sleeve and a distal sleeve), a snap component (encasing a sliding unidirectional component), a spring‐driven mechanism (including a thrust spring and a tension spring), a ratchet core rod, and a removable pre‐bent rod (Figure 1). The main sleeve is divided into two parts and connected via threading. The proximal titanium rod is connected to a rectangular core rod, which fits within the sleeve and provides anti‐rotation functionality. The snap component serves as a distraction distance marker, assisting the surgeon in observing the distraction distance in real time. The unidirectional sliding component achieves its function by contacting the ratchet on the core rod through an L‐shaped metal piece. The connection between the titanium rod and the rectangular core rod cannot pass through the proximal sleeve, thus acting as a stop to prevent slippage of the moving end. The spring‐driven mechanism acts on the core rod's head and base, providing dual internal driving forces to assist spinal growth. The removable pre‐bent rod is fixed to the distal end of the growing rod via screws, offering a replaceable rod option for EOS patients. The surgical methods for EOS are similar to those for the implantation of TGR. Clinical practitioners will need training to become proficient in the usage guidelines for the NGR.

### 3.2 Evaluation

After implantation into the body, growing rods undergo complex internal loads, and it has been reported that the device exhibits unfavorable mechanical responses under bending loads [44]. To assess whether the design meets strength requirements, finite element analysis (FEA) was conducted to simulate spinal bending loading conditions.

### 3.3 Subjects

**FIGURE 2:**
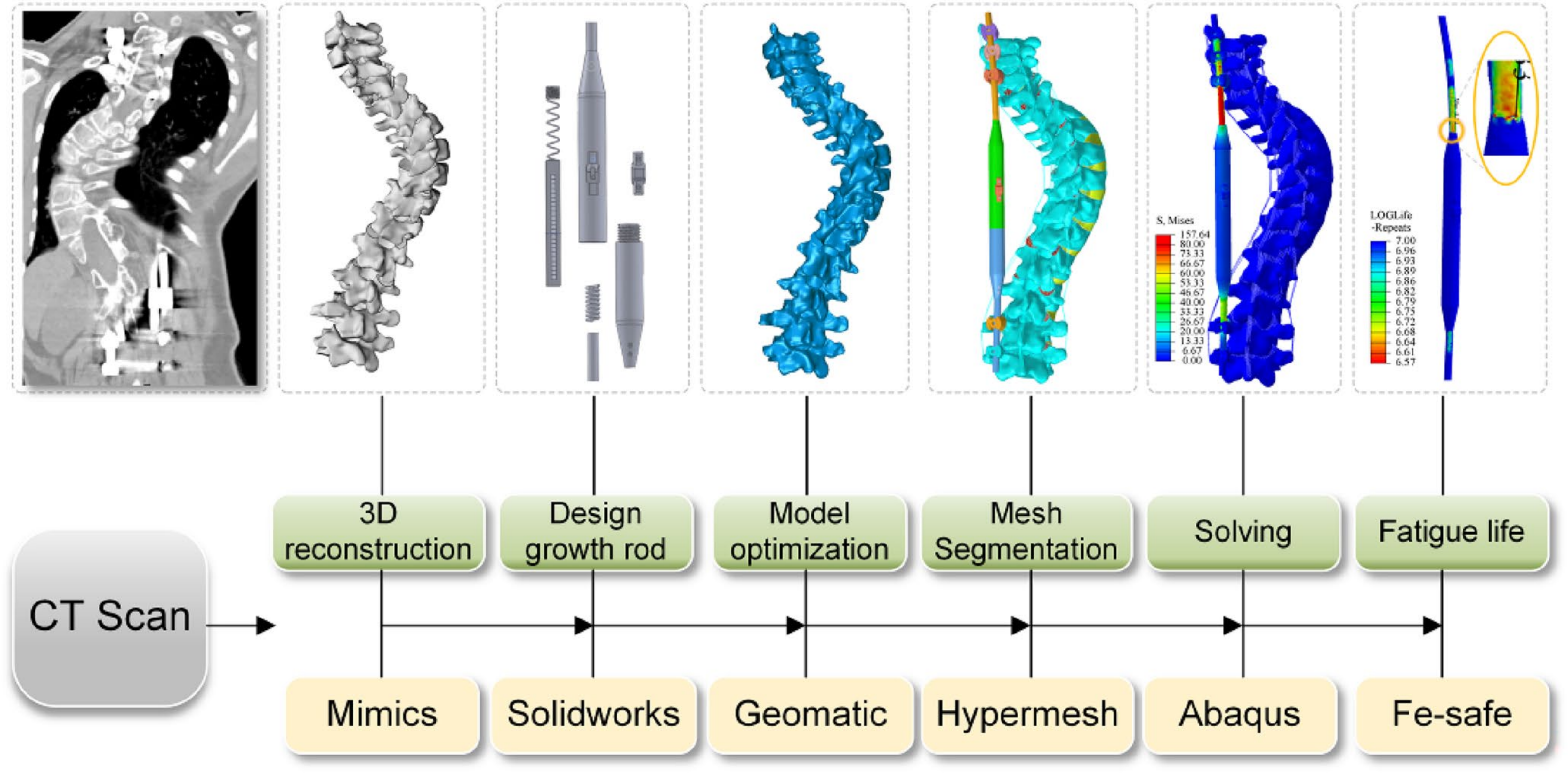
Experimental procedures.

The research indicates that the most common curve type is thoracic curvature [47]. The spine of a 12‐year‐old EOS patient was selected for analysis (Cobb angle: 47.79°). The patient's CT data was imported into Mimics (Materialise, Leuven, Belgium), where vertebral regions were individually delineated and the intervertebral boundary artifacts were eliminated. The surface was further smoothed in Geomagic Studio (Raindrop Geomagic Inc., Morrisville, NC), facilitating the model's conversion into a CAD surface model. The T2–L4 vertebral model was then imported into Solidworks (SolidWorks Corp., Waltham, MA). The T2–L4 vertebral model was then imported into Solidworks (Altair Engineering Inc., Troy, MI). Finally, the mesh models were imported into ABAQUS (Simulia, Providence, RI) software for material property assignment, loading, boundary condition application, and biomechanical analysis. Computational files were further processed in Fe‐safe (Simulia, Providence, RI) for fatigue life testing (Figure 2).

### 3.4 Adopted Mesh and Material Properties

Sequentially, material properties for each component of the spine and growing rod structures, including cortical bone, cancellous bone, endplate, intervertebral disc, facet joints, ligaments, and so on, were created (Table 1). Adult parameters were scaled proportionally to serve as pediatric spinal parameters [48]. All materials adhere to the basic assumptions of elasticity, including continuity, material homogeneity, isotropy of mechanical properties within the object, and linear elasticity.

**TABLE 1:** Material properties.

Unit composition	Unit type	*E* (MPa)	Poisson ratio	Cross‐sectional area (mm^2^)	Scaling factor	References
Cortical bone	Triangular shell	13 440	0.30	—	0.805^a^	[49]
Cancellous bone	Tetrahedron	241	0.30	—	0.805^a^	[50]
Endplate	Triangular prism	23.8	0.40	—	^b^	[51]
Nucleus pulposus	Tetrahedron	1	0.49	—	^b^	
Annulus fibrosus	Tetrahedron	4.2	0.45	—	0.782^a^	
ALL	Truss	7.8	0.12	63.7	0.893^a^	[51]
PLL		10	0.11	20.0		
TL		10	0.18	1.80		
CL		7.5	0.25	30.0		
ISL		8.0	0.14	30.0		
SSL		10	0.20	40.0		
LF		15	0.062	40.0		
Domino Connectors	Tetrahedron	110 000	0.30	—	—	[52]
Growing rods	Tetrahedron	110 000	0.30	—	—	
Screw	Tetrahedron	110 000	0.30	—	—	
Spring	Tetrahedron	20 700	0.29	—	—	[34]

^a^ The scaling factor for pediatric material parameters compared with adult material parameters [48].

^b^ The material parameters are the same as those of adults.

**FIGURE 3:**
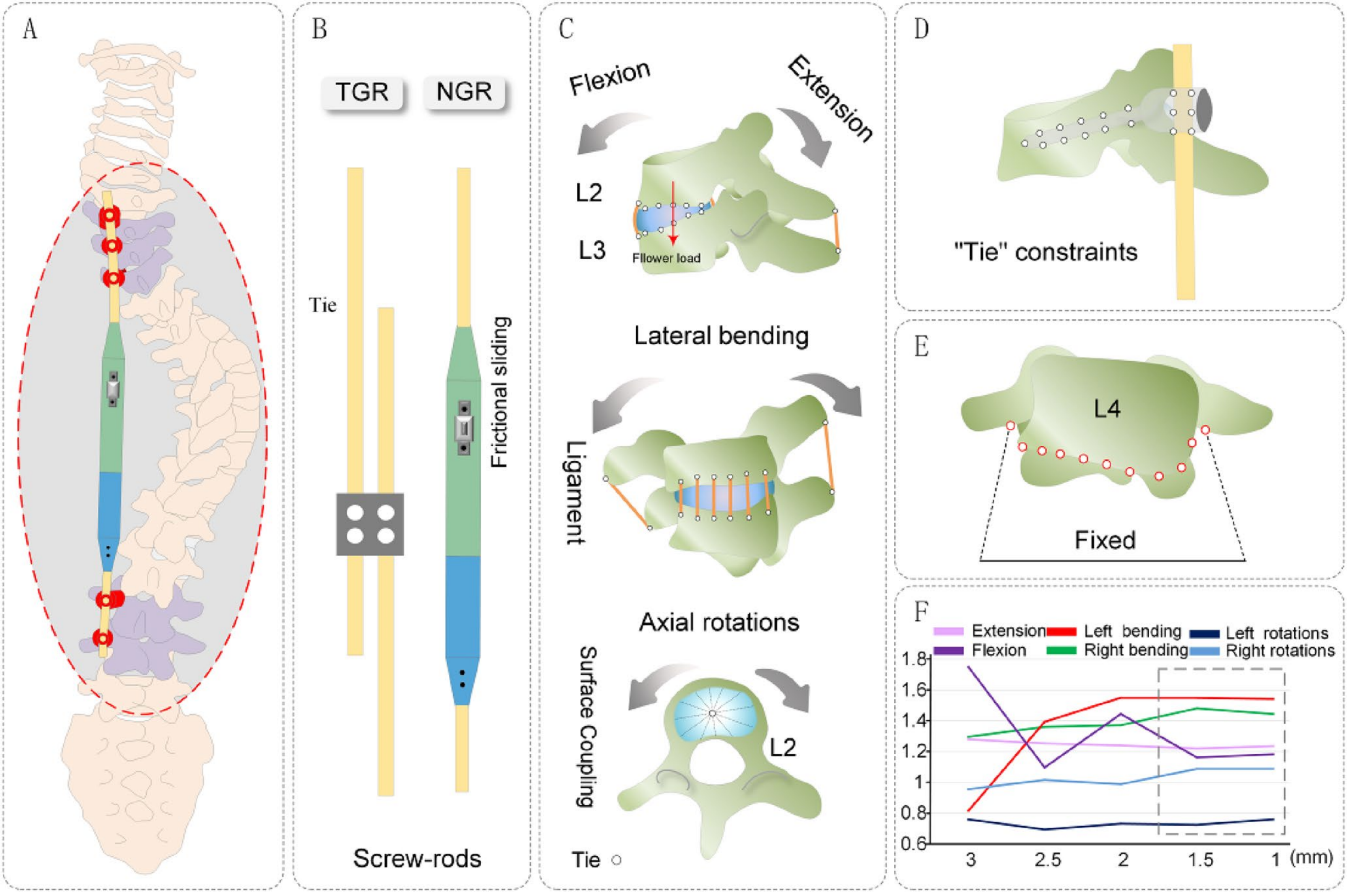
Boundary conditions, load setting, and mesh convergence analysis of models. (A) Backview; (B) TGR and NGR; (C) different conditions; (D) “Tie” constraints; (E) L4 fixation; (F) Mesh convergence analysis.

To ensure the accuracy and reliability of the model, a comprehensive mesh convergence analysis was conducted. Mesh sizes of 3, 2.5, 2, 1.5, and 1 mm were analyzed, and the results indicated that the test outcomes stabilized when the model's mesh size was < 2 mm (Figure 3F). In this study, an overall mesh size of 1.5–2 mm was adopted, with smaller mesh sizes chosen for the smaller components of the growing rod.

### 3.5 Boundary and Loading Conditions

**FIGURE 4:**
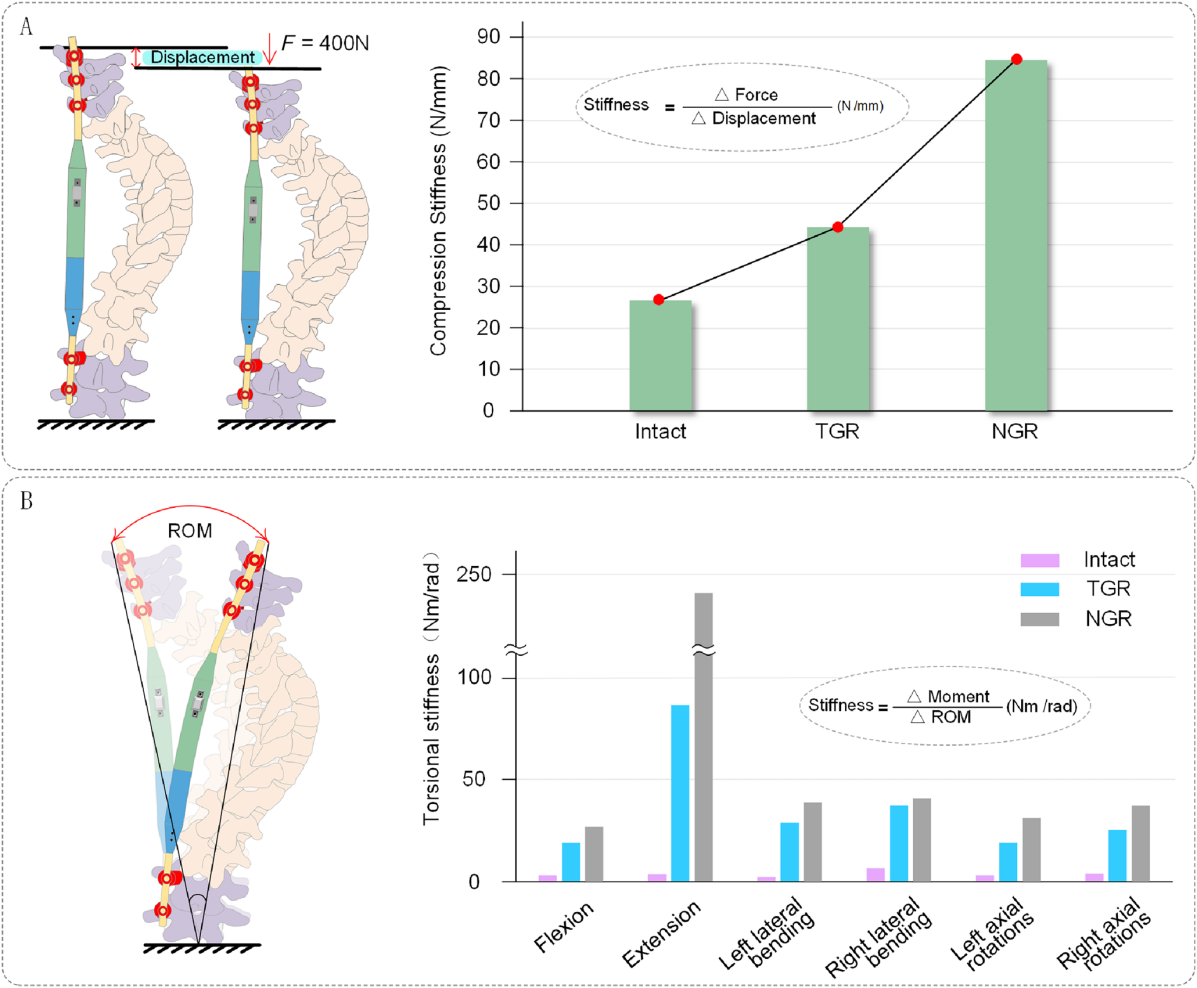
Stiffness of the growing rods. (A) Axial compression stiffness. (B) Torsional stiffness.

The articulating surfaces between facet joints were set as surface‐to‐surface contact with finite sliding and a friction coefficient of 0.1 [53]. Contact surfaces or points between the nucleus pulposus and annulus fibrosus, intervertebral disc and endplate, endplate and vertebral body, ligament and vertebral body, pedicle screw and vertebral body, growing rod and pedicle screw connector, and pedicle screw and growing rod were constrained with binding (Figure 3B–D) while sliding was allowed between the core rod and sleeve (Figure 3D). The following loads between vertebral bodies were set to simulate muscle forces (Table 2), with the loads applied along the center of the vertebral bodies. Fixing on the lower surface of L4 (*U* = UR = 0) (Figure 3E), while the upper surface of the T2 vertebra was subjected to a physiological load of 40 N (simulating the weight above the T2 segment) [54]. The load was applied vertically downward in the direction of gravity, accompanied by a bending moment of 1 NM [55] (Figure 3C). Additionally, a large load of 400 N was applied to the upper surface of the T2 vertebra to test its compressive stiffness [56] (Figure 4A).

**TABLE 2:** Follower load setting [57, 58].

Segment	Percentage of body weight (%)	Follower load (N)	Segment	Percentage of body weight (%)	Follower load (N)
T2	1.1	4.4	T9	1.6	6.4
T3	1.3 + 4.0 (Superior limbs)	21.2	T10	2.0	8
T4	1.3 + 4.0 (Superior limbs)	21.2	T11	2.1	8.4
T5	1.3 + 4.0 (Superior limbs)	21.2	T12	2.5	10
T6	1.3	5.2	L1	2.4	9.6
T7	1.4	5.6	L2	2.4	9.6
T8	1.5	6.0	L3	2.3	9.2

### 3.6 Fatigue Characteristics

The fatigue life analysis is conducted based on static analysis, where the results of the static analysis are imported into the Fe‐safe software. The software determines failure based on the stress amplitude and the expected number of cycles for fatigue failure, with a predefined cycle count > 10^7^ considered as failure. The stress and strain fields are obtained from Abaqus calculations. A bending moment ranging from 1 to 8 NM is set as a variable. The material parameters for the growing rod are sourced from the software material library, with Ti6Al4V selected and the “S–N Curve” algorithm employed, utilizing the maximum principal stress method (Table 3).

**TABLE 3:** Ti6Al4V parameters.

Material properties	
Ultimate tensile strength	1034 MPa
0.2% Yield stress	1006 MPa
*E*	120 350 MPa
Poissons ratio	0.33 MPa
Specimen type and orientation	Hourglass 6 mm dia polished
Failure criterion	0.5 mm crack length
Algorithm	PrincipalStrain
Stress–strain: *K*′	1702 MPa

## 4 Results

### 4.1 Stiffness of the Growing Rods

The axial compression stiffness of the NGR is significantly higher than that of the Intact group and the TGR group, with the TGR group exhibiting higher axial compression stiffness than the Intact group. The ranking of axial compression stiffness is as follows: NGR (84.44 N/mm) > TCR (44.36 N/mm) > Intact (26.76 N/mm) (Figure 4A).

In terms of torsional stiffness, it is observed that during flexion, the NGR value is 26.81 NM/rad, significantly higher than that of the Intact (3.07 NM/rad) and TGR (19.13 NM/rad) groups. During extension, the NGR value is 240.77 NM/rad, much higher than that of the Intact (3.76 NM/rad) and TGR (86.32 NM/rad) groups. In left bending, the NGR value is 38.90 NM/rad, significantly higher than that of the Intact (2.45 NM/rad) and TGR (28.88 NM/rad) groups. In right bending, the NGR value is 40.84 NM/rad, higher than that of the Intact (6.65 NM/rad) and TGR (37.32 NM/rad) groups. During left axial rotation, the NGR value is 31.11 NM/rad, significantly higher than that of the Intact (3.04 NM/rad) and TGR (19.15 NM/rad) groups. During right axial rotation, the NGR value is 37.24 NM/rad, significantly higher than that of the Intact (3.60 NM/rad) and TGR (27.51 NM/rad) groups (Figure 4B).

### 4.2 Spinal Joint ROM

**FIGURE 5:**
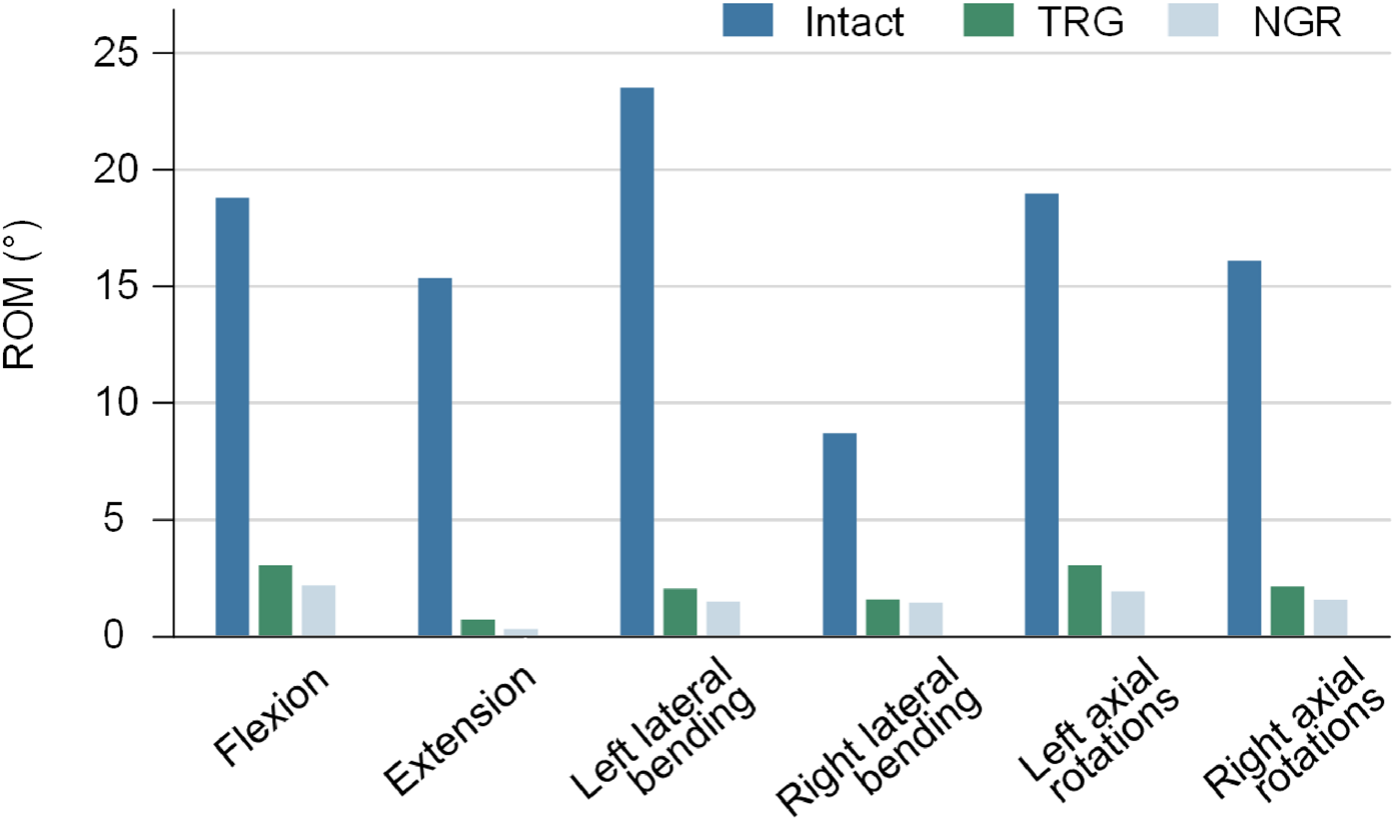
Spinal Joint ROM.

The ROM of NGR in the flexion conditions is 2.14°, significantly lower than TGR's 3.00° and Intact's 18.66°. In the extension, the ROM of NGR is 0.24°, much lower than TGR's 0.66° and Intact's 15.22°. In the left bending, the ROM of NGR is 1.47°, less than TGR's 1.98° and Intact's 23.35°. In the right bending, the ROM of NGR is 1.40°, lower than TGR's 1.54° and Intact's 8.62°. In the left axial rotation, the ROM of NGR is 1.84°, significantly lower than TGR's 2.99° and Intact's 18.85°. Finally, in the right axial rotation conditions, the ROM of NGR is 1.54°, lower than TGR's 2.08° and Intact's 15.93° (Figure 5).

### 4.3 The Distribution of Von Mises Stress in Vertebrae and Intervertebral Discs

**FIGURE 6:**
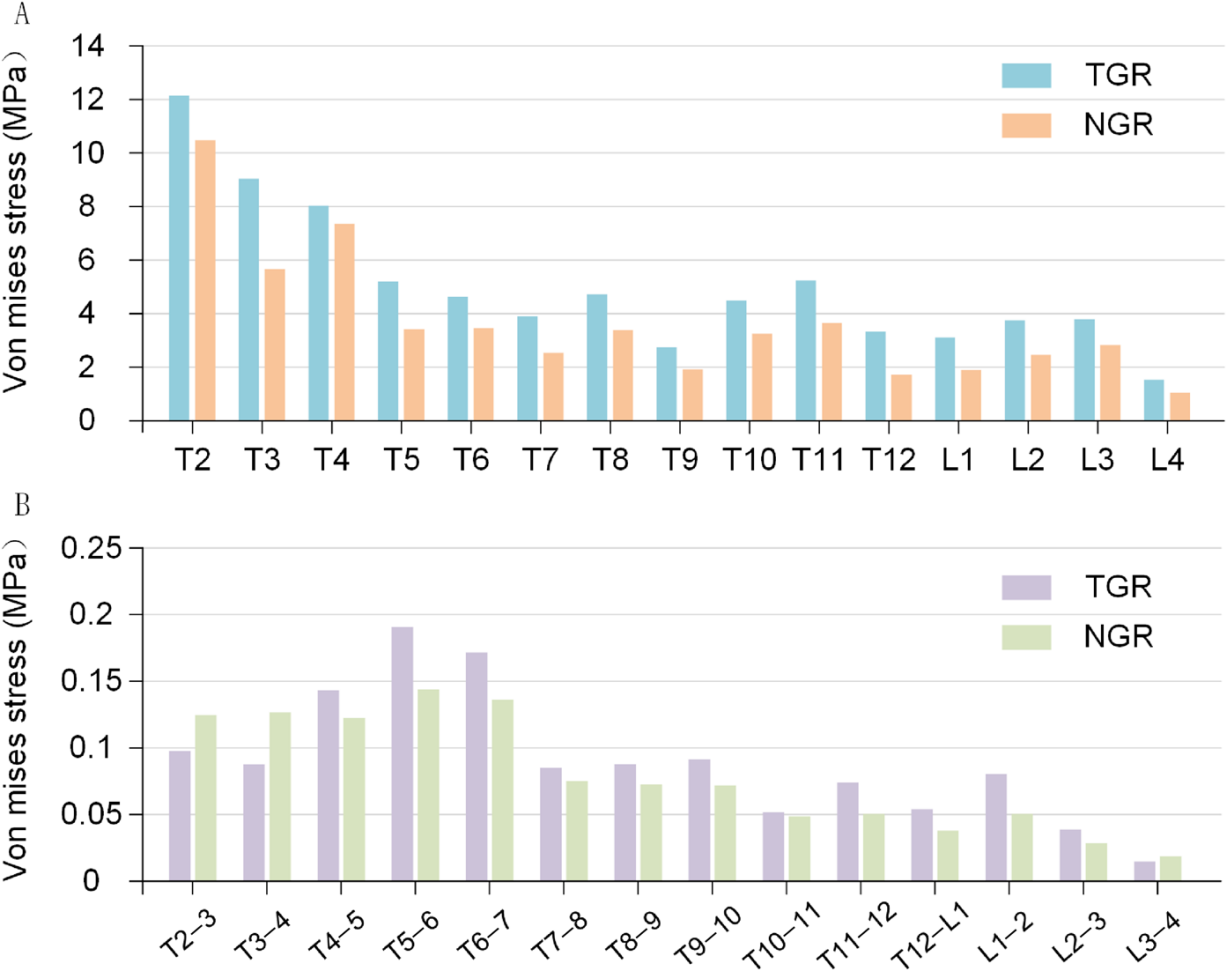
The distribution of Von Mises stress in vertebrae and intervertebral discs. (A) Stress distribution in vertebrae. (B) Stress distribution in intervertebral discs.

TGR: The peak intervertebral disc stress is concentrated at the T5–6 and T6–7 segments (0.19 and 0.17 MPa, respectively). The lowest stress occurs at the L3–4 segment, measuring only 0.01 MPa. Overall, NTR: Compared with TGR, the intervertebral disc stress in NTR is reduced. The maximum stress occurs at the T2–3 segment, measuring 0.12 MPa. The lowest stress is observed in the T12–L1 and L3–4 regions (0.04 and 0.02 MPa, respectively) (Figure 6A). TGR: The peak vertebral stress is concentrated at T2 (12.14 MPa), with stress gradually decreasing as the vertebrae extend downward. In the T3–T6 region, stress diminishes to 5.20 MPa as the vertebrae descend, and lower stress values are also noted at T7–T9. Stress is more concentrated in the T11–L3 region, particularly at the T11 vertebra, where it reaches 5.24 MPa. NTR: The peak vertebral stress also appears at T2 (10.48 MPa), but this value is lower than that of TGR. Additionally, the stress in the bending region of NTR is generally lower than that of TGR. In the T3–T9 region, the stress in the NTR system is lower, especially near T9, where the value drops to 1.92 MPa (Figure 6B).

### 4.4 The Distribution of Von Mises Stress in the Growing Rod

**FIGURE 7:**
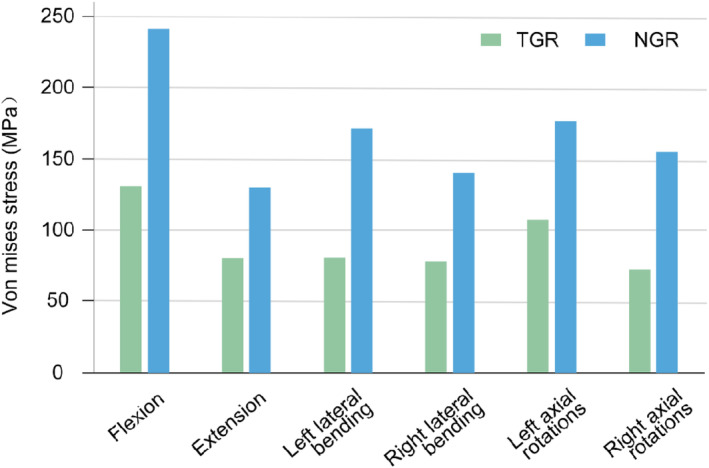
The Von Mises stress values for the growing rod.

**FIGURE 8:**
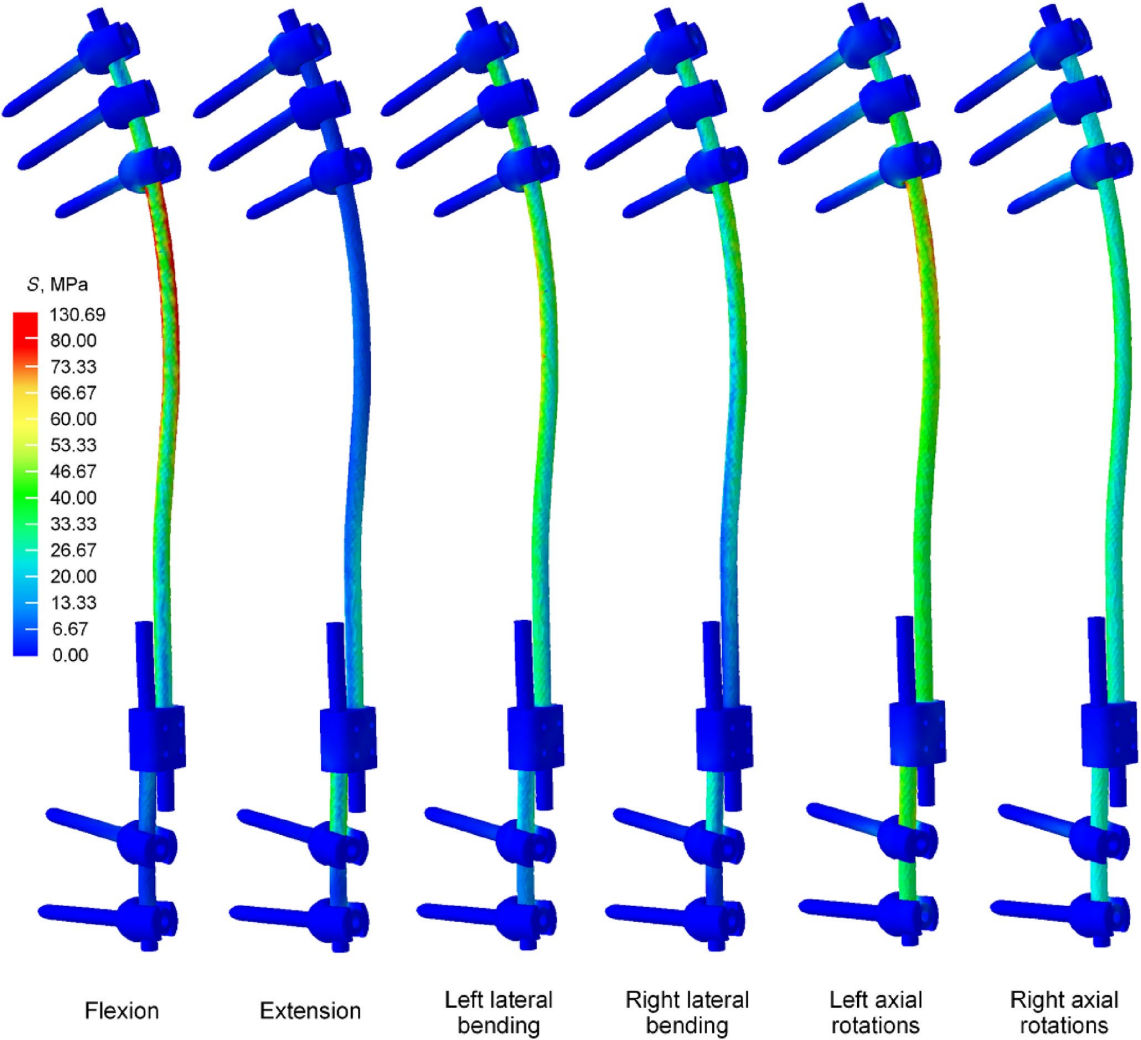
The Von Mises stress distribution for the TGR.

**FIGURE 9:**
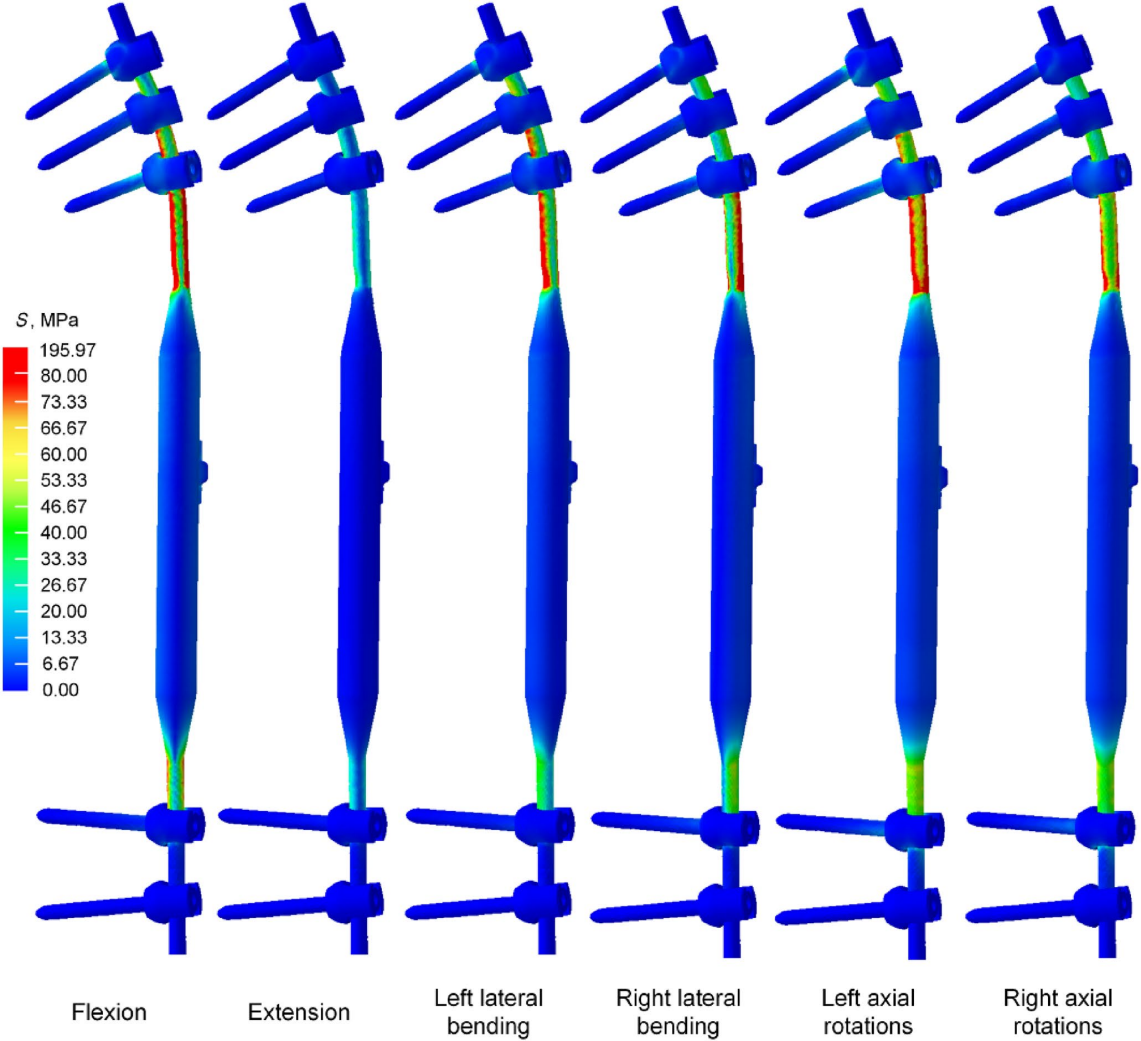
The Von Mises stress distribution for the NGR.

In the flexion conditions, the Von Mises stress for NGR is 241.75 MPa, significantly higher than that of TGR (130.69 MPa). In the extension conditions, the Von Mises stress for NGR is 130.24 MPa, higher than TGR's (80.20 MPa). In the left bending conditions, the Von Mises stress for NGR is 170.99 MPa, considerably higher than TGR's (80.45 MPa). In the right bending conditions, the Von Mises stress for NGR is 140.72 MPa, higher than TGR's (77.96 MPa). In the left axial rotation conditions, the Von Mises stress for NGR is 176.81 MPa, significantly higher than TGR's (107.23 MPa). Finally, in the right axial rotation conditions, the Von Mises stress for NGR is 155.77 MPa, considerably higher than TGR's (77.72 MPa) (Figure 7). From the Von Mises stress contour plot, it is observed that the Von Mises stress for both NGR and TGR mainly concentrates near the locations where the screws are fixed at both ends (Figures 8 and 9).

### 4.5 The Axial Force and Bending Moment by the Growing Rod

**FIGURE 10:**
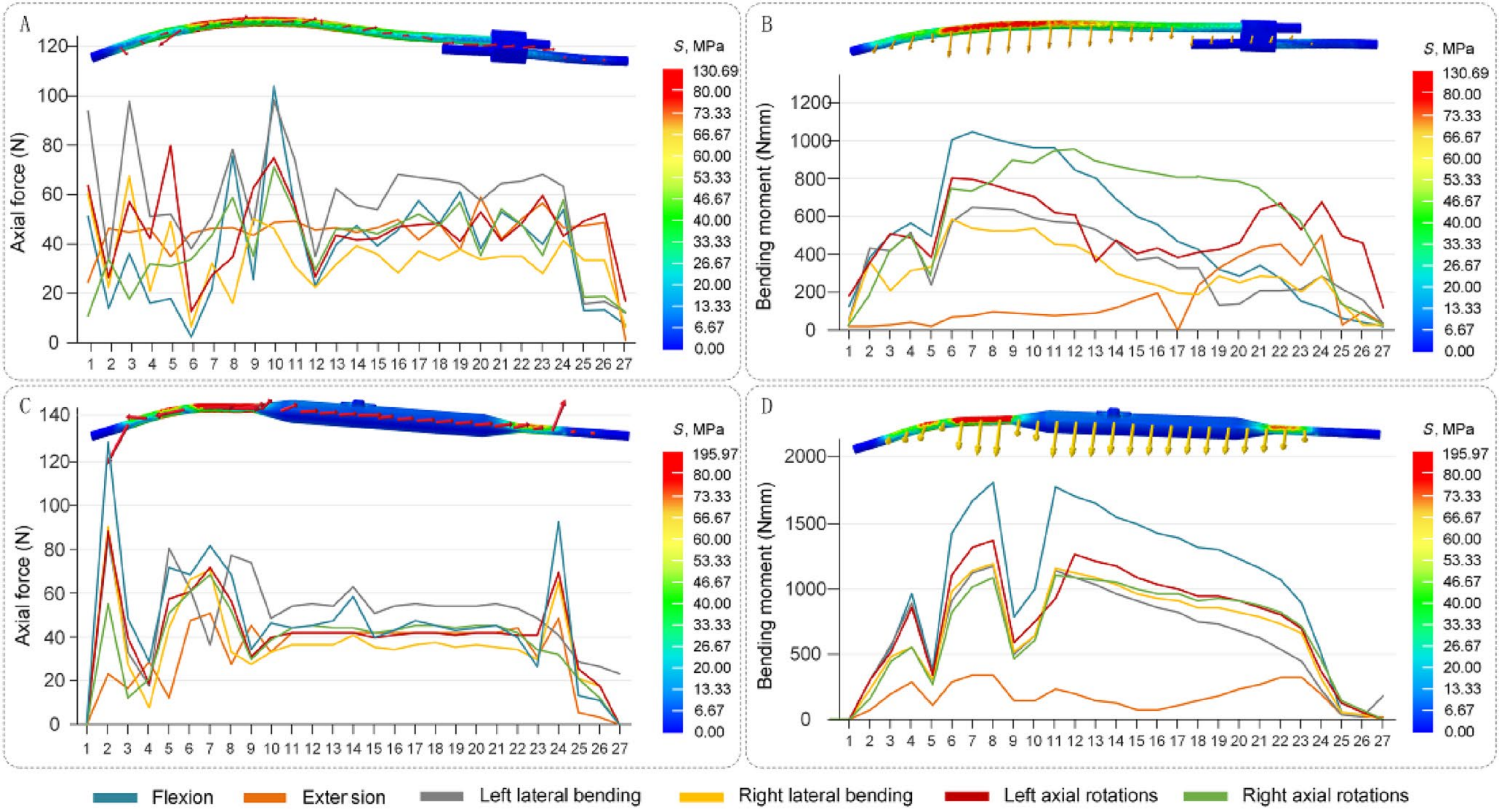
The axial force and bending moment values of the growing rod. (A) TGR axial force values. (B) TGR bending moment values. (C) NGR axial force values. (D) NGR bending moment values.

Slicing analysis of the growing rod was performed to analyze axial force and bending moment. In various motion directions, there are differences in the maximum and average axial forces between TGR and NGR. In flexion and right bending conditions, the maximum axial force of NGR (127.90 N, 90.18 N) is greater than that of TGR (104.10 N, 67.79 N), while in other conditions, the maximum axial force of NGR is slightly less than TGR. The maximum axial forces of TGR are mainly concentrated near the upper screw fixation, while those of NGR are concentrated near both the upper and lower screw fixations. Observing the average axial force of the growing rod, it is noted that in the left bending condition, the average axial force of TGR and NGR is the highest (57.80 N, 49.41 N) (Figure 10A,C).

In all motion directions, the bending moment of NGR is greater than TGR. In the flexion condition, the maximum bending moment of TGR and NGR is the highest (1086 and 1052 Nmm). Observing the average bending moment of the growing rod, it is found that in the extension condition, the bending moment of NGR is slightly higher than TGR (169.11 and 154.29 Nmm), while in other conditions, the bending moment of NGR is significantly higher than TGR. The higher bending moments of TGR and NGR are mainly concentrated in the upper part of the growing rod (Figure 10B,D).

### 4.6 Fatigue Life of the Growing Rod

For both TGR and NGR, in all conditions with bending moments ranging from 1 to 3 NM, the fatigue life exceeds 10^7^ cycles, and no damage is observed (Table 4). In the extension condition with bending moments ranging from 1 to 3 NM, the fatigue life of both TGR and NGR exceeds 10^7^ cycles, with no damage observed. Even under a 4 NM bending moment, both TGR and NGR still exhibit good fatigue life. However, for bending moments ranging from 5 to 8 NM, there is a decreasing trend in the fatigue life of the growing rod, with the performance of TGR being superior to that of NGR.

**TABLE 4:** The fatigue life of the growing rod under various conditions with different bending moments (cycles).

	Flexion	Extension	Left lateral bending	Right lateral bending	Left axial rotations	Right axial rotations
TGR						
1 NM	> 10^7^	> 10^7^	> 10^7^	> 10^7^	> 10^7^	> 10^7^
2 NM	> 10^7^	> 10^7^	> 10^7^	> 10^7^	> 10^7^	> 10^7^
3 NM	> 10^7^	> 10^7^	> 10^7^	> 10^7^	> 10^7^	> 10^7^
4 NM	1 697 645.88	> 10^7^	> 10^7^	> 10^7^	> 10^7^	> 10^7^
5 NM	251 813.48	> 10^7^	4 333 822.00	> 10^7^	> 10^7^	> 10^7^
6 NM	63 373.70	> 10^7^	878 204.63	2 776 650.50	3 165 803.25	3 737 518
7 NM	23 766.25	> 10^7^	242 497.92	724 327.63	822 716.38	1 310 715.5
8 NM	11 543.69	> 10^7^	87 571.92	238 593.72	268 832.25	522 356.906
NGR						
1 NM	> 10^7^	> 10^7^	> 10^7^	> 10^7^	> 10^7^	> 10^7^
2 NM	> 10^7^	> 10^7^	> 10^7^	> 10^7^	> 10^7^	> 10^7^
3 NM	> 10^7^	> 10^7^	> 10^7^	> 10^7^	> 10^7^	> 10^7^
4 NM	578 180.875	> 10^7^	> 10^7^	2 431 216.25	5 612 971.5	> 10^7^
5 NM	61 429.52	> 10^7^	983 080.188	196 837	438 444	258 026.297
6 NM	15 761.435	> 10^7^	151 098.266	37 934.609	74 172.039	45 068.43
7 NM	6358.163	> 10^7^	40 856.73	13 352.099	22 822.824	15 058.384
8 NM	3196.998	> 10^7^	16 456.23	6497.649	10 158.198	7288.339

**FIGURE 11:**
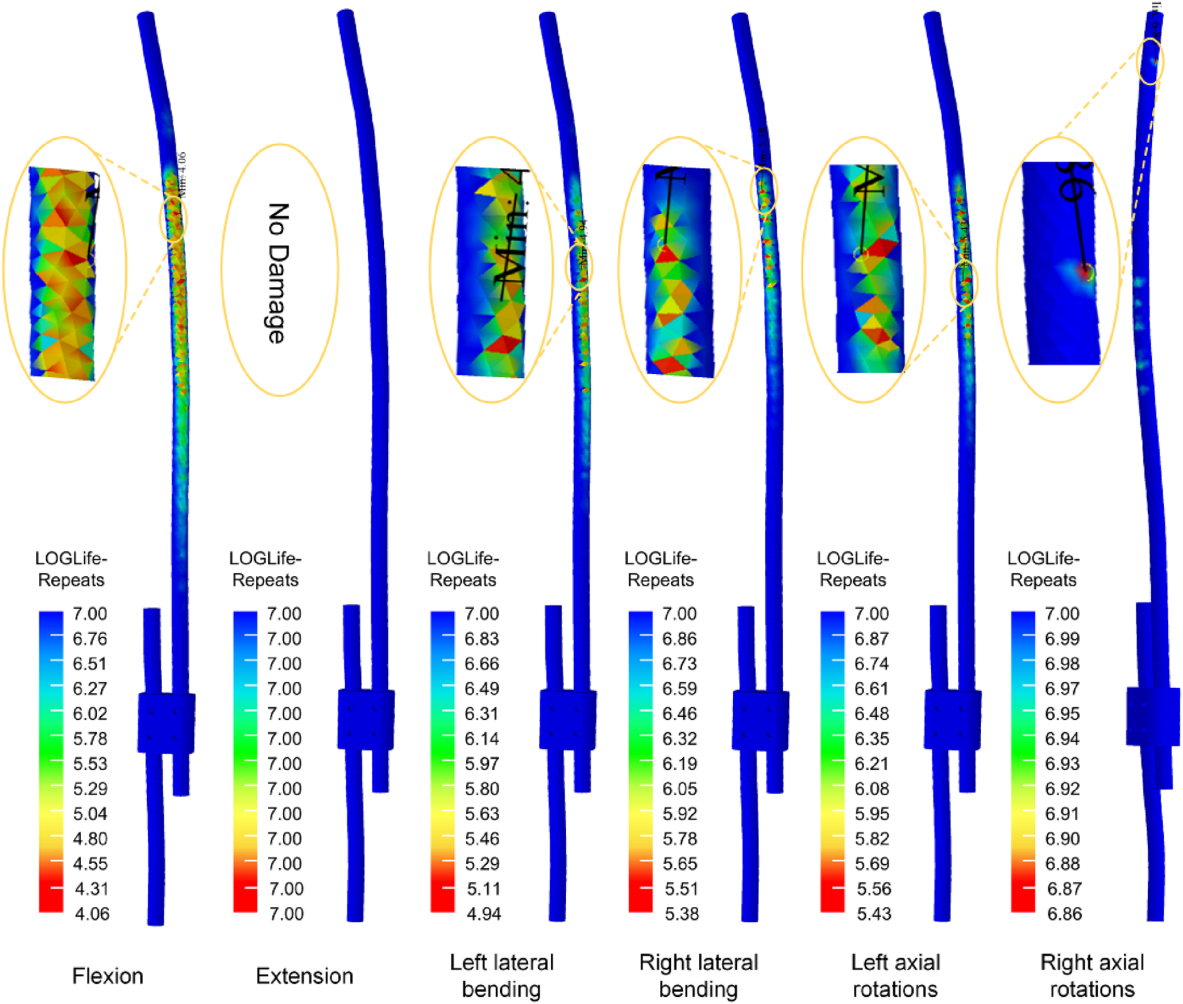
The fatigue life plot for TGR.

**FIGURE 12:**
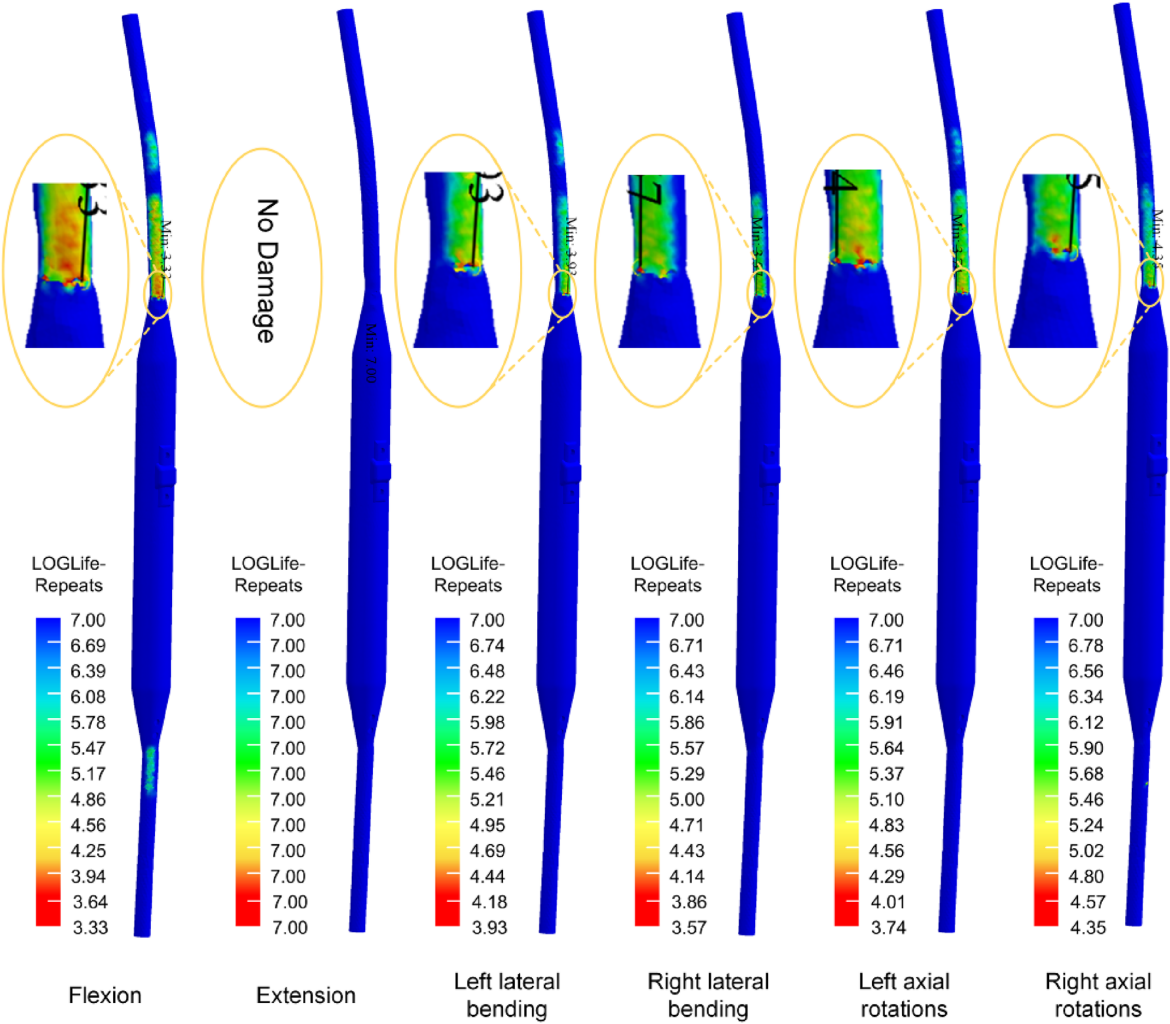
The fatigue life plot for NGR.

From the fatigue life contour plots, it can be observed that under the extension condition, TGR shows no fatigue damage. In other conditions, fatigue damage first appears near the upper screw fixation position. For NGR, under the extension condition, no fatigue damage is observed. However, in other conditions, fatigue damage first appears at the exit position where the core rod slides out of the sleeve (Figures 11 and 12).

### 4.7 Strain Energy of the Growing Rod

**FIGURE 13:**
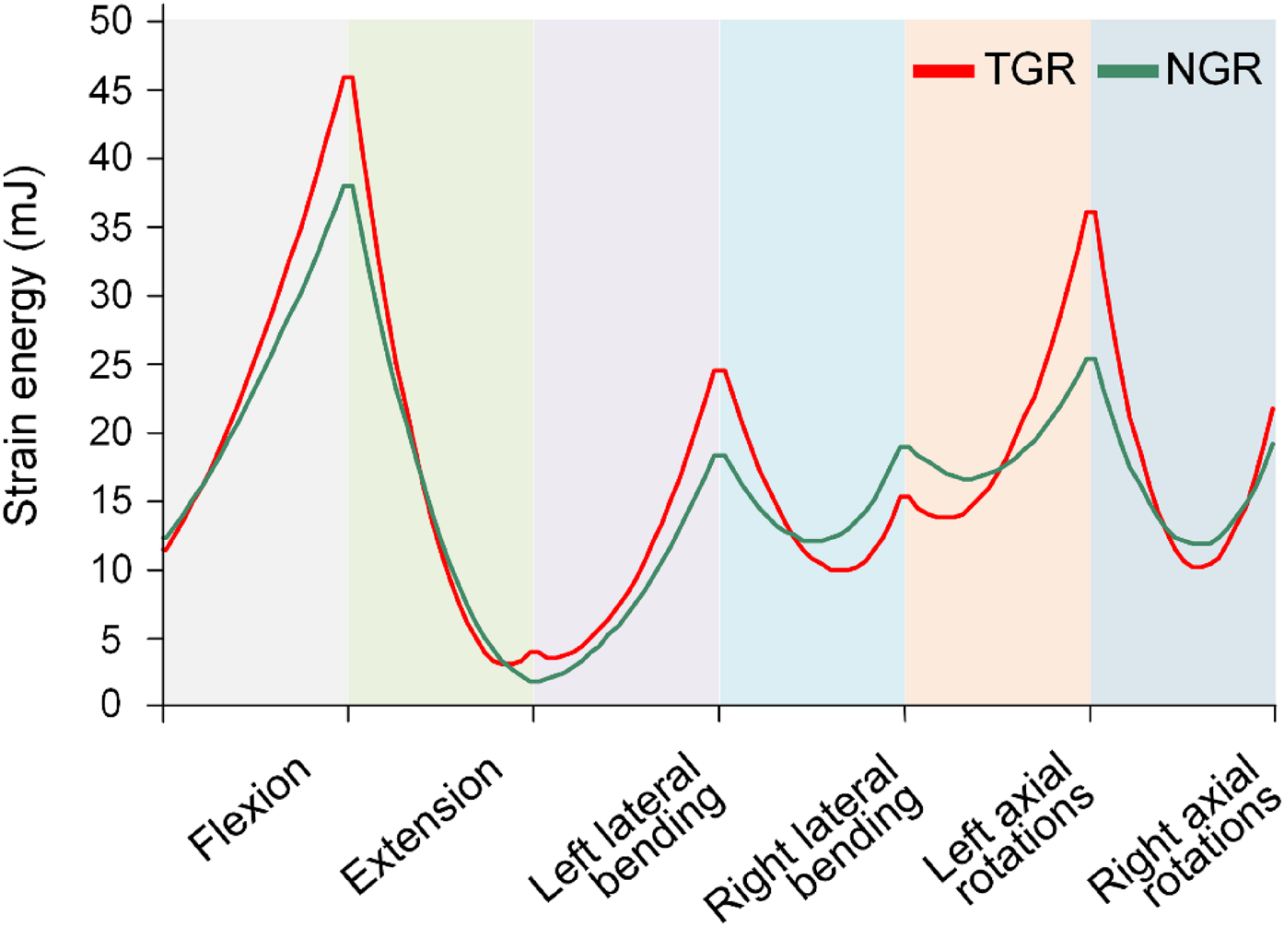
The strain energy of the growing rod system.

Strain energy is the energy stored in a component due to elastic deformation (Figure 13). In flexion–extension conditions, the maximum strain energy for TGR and NGR is 46.12 and 38.05 mJ, respectively. In lateral bending conditions, the maximum strain energy for TGR and NGR is 24.57 and 18.40 mJ, respectively. In rotation conditions, the maximum strain energy for TGR and NGR is 36.06 and 25.43 mJ, respectively. In lateral bending conditions, the strain energy of TGR and NGR is greater than in other conditions. Compared with TGR, NGR exhibits smaller fluctuations in strain energy across all conditions, and the overall strain energy of NGR is less than that of TGR (Figure 13).

## 5 Discussion

The study describes the development process of an NGR, including design and mechanical performance evaluation. The design of this device aims to address the complications reported with current devices used for EOS surgical treatment. FEA was conducted to design and analyze the implant, predicting its mechanical characteristics under extreme operating conditions. Compared with TGR, NGR exhibits significantly increased compression and torsional stiffness, indicating improved structural rigidity. NGR demonstrates reduced spinal joint ROM in all directions compared with TGR and Intact, suggesting enhanced fixation properties, especially in extension. After implantation of the growing rod, spinal joint ROM decreases notably, with NGR experiencing a more pronounced decrease. Stress on the pedicle screws is significantly higher with NGR in all directions compared with TGR, indicating superior performance in load‐bearing and stabilization. However, this also implies that NGR may require higher material strength and design optimization in practical applications to ensure long‐term reliability and stability under high‐stress conditions. Stress tends to concentrate near the screw fixation locations.

In the analysis of solid mechanics, it is often necessary to analyze the internal forces of a solid component to study its mechanical performance. To investigate the magnitude of internal forces in each segment of a rod under external loads, it is common to determine the internal force diagram (axial force and bending moment) of the rod. This provides a design basis for strength and stiffness designs by identifying the locations of critical sections. Components with higher axial forces are more prone to damage, requiring materials with greater strength to support them. In conditions such as extension, left bending, and left–right rotation, the axial forces in NGR are smaller than those in TGR, indicating superior performance of NGR in these conditions. A higher bending moment represents better bending and rotational performance of the device. In terms of bending moment, NGR consistently exhibits higher values than TGR, indicating good bending and rotational performance. Larger bending moments help distribute and transfer loads, ensuring structural stability and strength. In some cases, larger bending moments can increase structural stiffness and resistance to bending, reducing structural deflection. Under the premise of ensuring structural safety, larger bending moments can sometimes optimize material utilization. NGR provides better structural stiffness, resulting in a reduction in the ROM of the spine after implantation, showing a negative correlation between structural stiffness and ROM. From a theoretical perspective, the amount of fracture material generated during the failure process of a solid structure should be related to the strain energy in the solid before failure. Compared with TGR, NGR exhibits smaller strain energy, which may suggest better energy absorption during bending. Consequently, NGR may have better bending performance. Shu and Fu also identified reducing mechanical strain energy through multiple optimizations of the connecting rods as one of the indicators [59].

When the structure of an implant is excessively rigid, it can induce stress‐shielding effects in the surrounding tissues. In post‐EOS surgery, the spine often experiences extreme tensile stress, significantly affecting it. In this study, compared with TGR, the stress on both the vertebrae and intervertebral discs is reduced with the NGR. Many may question whether the NGR could lead to significant stress shielding and accelerate bone quality loss. However, the NGR exhibits a reduced stress shielding effect due to the substantial tensile forces exerted on the spine. As the spine grows, the internal tensile forces gradually diminish and transform into compressive forces. This implies that patients implanted with TGR may require extension surgery before the end of their growth period. NGR can maintain the spine under tension through short‐term outpatient traction. Rohlmann et al. [60] conducted a study involving five participants, measuring ~1000 different activities and parameters of torque. The results revealed that during shoe‐wearing activities, the maximum torque reached 3.5 Nm, whereas in other daily activities, the torque typically remained below 3 Nm. Under low torque conditions (1–3 Nm), both NGR and TGR exhibited excellent performance, with prolonged fatigue life (exceeding 10^7^ cycles), capable of meeting the demands of daily life. However, under high torque conditions, the fatigue life of both NGR and TGR was reduced. Nevertheless, they still maintained a high fatigue life under various torque conditions, demonstrating greater adaptability and stability. Therefore, NGR may be a reliable and adaptable choice for skeletal growing rods, particularly suitable for situations requiring high loads and extreme conditions. Regarding TGR, the location of the first appearance of fatigue damage was near the titanium rod close to the upper fixed screw. The first signs of fatigue in NGR are observed at the location where the core rod slides out of the sleeve exit. To enhance NGR, it is imperative to reinforce the strength at this location. During flexion and lateral bending, the strain energy of TGR is slightly higher than that of NGR, suggesting that TGR may experience some deformation or greater energy loss during bending. In contrast, NGR may demonstrate superior bending performance. Under extension and axial rotation conditions, the strain energy of both TGR and NGR is essentially similar, indicating that these two types of growing rods perform similarly under tensile stress. A common and serious complication in EOS treatment is rod breakage, which has been the focus of several studies [44]. The structural arrangement and applied loads govern elevated bending forces at three specific junctures within the structure: (1) at the central juncture, (2) neighboring the linked connector, or (3) neighboring the anchoring base at the far end [44, 61]. Material longevity may be influenced by four key factors: Shot‐peening, a well‐known method to induce surface compressive stress, serves as a renowned delay mechanism for fatigue crack initiation. Pre‐bending of titanium rods impacts local stress distribution. The generation of surface defects such as indentations. Accumulation of load cycles, that may result in the nucleation and propagation of fatigue cracks. Croonenborghs et al. [62] have shown that enhancing residual stresses in the near‐surface region can extend fatigue life. This study predicts critical stress locations on growing rods akin to those observed in vivo failures, providing a fundamental assessment of spinal implant strength to ensure device stability.

Currently, a few researchers are also dedicated to improving or designing new types of growing rods. Li et al. have developed a multisegment growth guidance rod for operations in small animal models, focusing on its elongation effects and device failure rates, and have preliminarily validated the feasibility of a unidirectional elongation growing rod [63]. Shekouhi et al. explored the impact of different parameters (such as axial connector configuration, traction frequency, and elongation magnitude) on the fracture rate of growing rods and proposed a new device suitable for long‐growing rods [34]. Holewijn et al. [64] a posterior concave periapical distraction device for fusionless scoliosis correction was introduced, and a more physiological spinal motion is expected after scoliosis correction with the posterior concave periapical distraction device.

Rana et al. [65] and Biswas et al. [66] propose that spinal instrumentation treatments require continuous development of advanced flexible or semi‐rigid rod geometries to achieve better clinical outcomes. Recent studies have begun to explore the use of springs as auxiliary driving forces to promote the growth of growing rods, with some clinical trials showing promising results [67]. Currently, there are some deficiencies in growing rod technologies available on the market, but there is still significant design space. However, careful consideration of the economic feasibility is necessary if the complexity of the device increases. As the structural complexity of the device increases, so does the cost, along with an increased risk of failure. In the future, new types of growing rods should possess the following characteristics: first, the structure should conform to the spinal curve, while improving lung function and quality of life should be considered as primary goals. Second, growing rods that can be adjusted externally are urgently needed in clinical practice, with research focusing on methods such as magnetic control, chip control, or manual traction adjustment. And finally, growing rods should assist spinal growth, achieving the essence of “growth” through the principle of unidirectional sliding.

**FIGURE 14:**
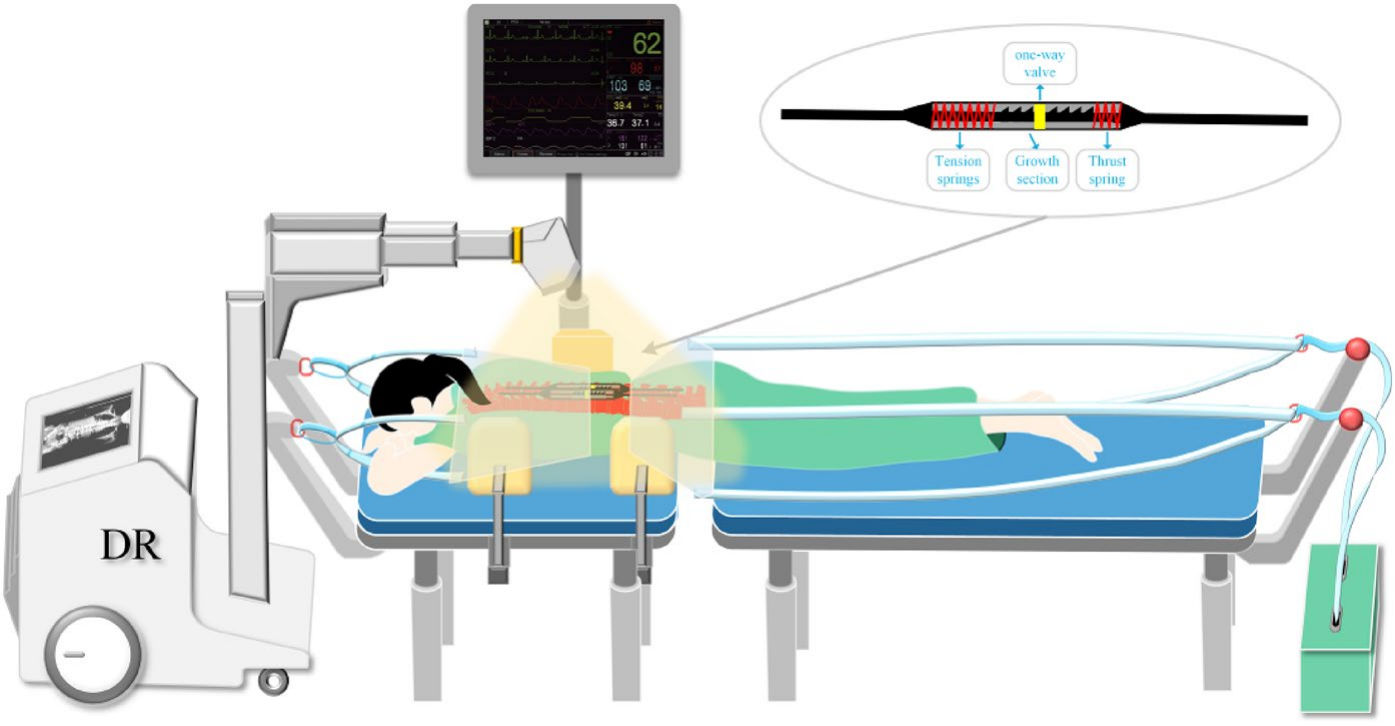
Future concepts: EOS precision traction rod tuning outpatient therapy.

The importance of surgery in EOS treatment is self‐evident. Surgeons continue to seek optimization of patient treatment plans. An ideal management approach entails a single implantation surgery, avoiding multiple extension surgeries, limiting the anatomical scope to ensure maximal ROM and prevent fusion, preserving sufficient mobility to reduce fatigue and rod breakage risks, easily shaping contours, and promoting spinal growth through continuous bending. By delaying the age of initial rod implantation, using dual rods, and submuscular implantation, and limiting the number of surgeries, surgical complications can be reduced, and the need for additional surgeries lowered. While braces are suitable for early‐stage patients, new postoperative flexible braces and specific traction beds for patients with implanted growing rods are expected to improve treatment outcomes further. Potential new approaches include implanting spring‐driven unidirectional limit growing rods, wearing flexible braces postoperatively to resist lateral forces and assist spinal correction, and adjusting every 2 months in the clinic using a special spinal curvature traction bed. The surgical method still follows the traditional implantation technique of TGR. To prevent the occurrence of over‐distraction, EOS treatment necessitates multiple small‐distance distractions. In clinical practice, the majority of cases utilize bilateral growing rod techniques, with future experiments planned to validate the dual‐rod system. Postoperative trunk imbalance frequently occurs, requiring surgical asymmetrical distraction for correction. Future research will concentrate on methods for controlled external adjustment of this growing rod. This bed employs head‐to‐foot axial traction, a three‐point lateral corrective push plate, and a fluoroscopy machine to accurately adjust rod position, with internal unidirectional limit devices maintaining the current state, achieving non‐invasive, precise, fluoroscopy‐guided rod adjustments (Figure 14). Referring to the specific clinical complications of Magnetically MCGR, the NGR in this study may also lead to the deposition of metal fragments affecting adjacent soft tissues, as well as changes in serum metal ion concentrations. To address similar issues, two solutions will be proposed in future studies: (1) developing superior materials or specialized coatings, and (2) designing a foldable biomembrane on the surface of the sliding sleeve to separate metal debris from tissues. These new approaches may improve patient quality of life, but further research is needed to validate and develop treatment guidelines (Figure 14).

The limitation of this study lies in its focus on testing the stability of NGR without delving into its sliding growth functionality. Considering the body's exposure to multidirectional loads in real loading environments, this complexity was not fully addressed in the study. This study simplified the complex physiological environment and material properties of the human body and did not simulate the anisotropic material properties of various tissues. Future research should refine the model's material properties to explore more valuable results. The influence of the thoracic cavity has a certain impact on the correction of EOS; however, this study has overlooked the thoracic cavity. Therefore, we will conduct a more in‐depth exploration of the thoracic cavity's effects in future studies. Future research will prioritize the fabrication of NGR and conduct in vitro mechanical performance tests to assess its potential wear, the effect of hardware pull‐out and growth adjustment characteristics, further confirming its prospects for clinical application. In the future, clinical data support will be essential for the NGR to realize its full potential.

## 6 Conclusions

This study introduces a novel dynamic implant device designed to correct three‐dimensional spinal deformities in EOS patients. The device utilizes a structure designed based on the principle of distraction, comprising unidirectional sliding components and a spring‐driven mechanism, enabling dynamic adjustment functionality. Additionally, it introduces an innovative non‐invasive extension mechanism to reduce the risk of infection. Through FEA for design and evaluation, the new device demonstrates higher stability compared with the currently dominant EOS implant devices in the market. This research provides a new equipment option that may contribute to optimizing EOS surgical treatment strategies, with the potential to enhance the efficacy of EOS surgical treatment. More importantly, this study proposes innovative treatment strategies, paving new pathways for the treatment of EOS.

## Ethics Statement

The research was approved by the Science and Ethics Committee of the School of Biological Science and Medical Engineering at Beihang University (protocol code: BM20240037).

## Conflicts of Interest

The authors declare no conflicts of interest.
